# Naringin improves random skin flap survival in rats

**DOI:** 10.18632/oncotarget.21589

**Published:** 2017-10-06

**Authors:** Liang Cheng, Tingxiang Chen, Qiming Tu, Hang Li, Zhenghua Feng, Zhijie Li, Dingsheng Lin

**Affiliations:** ^1^ Department of Hand and Plastic Surgery, The Second Affiliated Hospital and Yuying Children’s Hospital of Wenzhou Medical University, Wenzhou, Zhejiang, China

**Keywords:** naringin, random flap survival, angiogenesis, inflammation, rat

## Abstract

**Background:**

Random-pattern flap transfer is commonly used to treat soft-tissue defects. However, flap necrosis remains a serious problem. Naringin accelerates angiogenesis by activating the expression of vascular endothelial growth factor (VEGF). In the present study, we investigated whether naringin improves the survival of random skin flaps.

**Results:**

Compared with controls, the naringin-treated groups exhibited significantly larger mean areas of flap survival, significantly increased SOD activity and VEGF expression, and significantly reduced MDA level. Hematoxylin and eosin (HE) staining revealed that naringin promoted angiogenesis and inhibited inflammation.

**Materials and Methods:**

“McFarlane flap” models were established in 90 male Sprague-Dawley (SD) rats divided into three groups: a 40 mg/kg control group (0.5 % sodium carboxymethylcellulose), a 40 mg/kg naringin-treated group, and an 80 mg/kg naringin-treated group. The extent of necrosis was measured 7 days later, and tissue samples were subjected to histological analysis. Angiogenesis was evaluated via lead oxide–gelatin angiography, immunohistochemistry, and laser Doppler imaging. Inflammation was evaluated by measurement of serum TNF-α (tumor necrosis factor-α) and IL-6 (interleukin-6) levels. Oxidative stress was assessed by measuring superoxide dismutase (SOD) activity and the malondialdehyde (MDA) level.

**Conclusion:**

Naringin improved random skin flap survival.

## INTRODUCTION

Flaps are commonly used to remedy cosmetic defects, restore function after traffic accidents and other traumas, and in the aged. Aydin and Liang W used pedicled, nasolabial island flaps to rebuild the nose after removal of nose-tip basal cell carcinomas [1, 2]. Free flap reconstruction has also been employed to treat breast cancer patients [3]. Morris used a perforator flap to repair a large defect on the back [4]. Random skin flaps that are similar to the wound in texture, color, and dermatoglyphic properties are often employed to repair small soft-tissue defects evident after tumor resection. An adequate blood supply is essential for flap survival; the possibility of necrosis (especially complete necrosis) must be considered when designing and placing flaps [5]. Many agents have been reported to improve flap survival by inhibiting inflammation, increasing free-radical scavenging, promoting angiogenesis, and/or expanding the microvascular network. Such materials include VEGF [6], sildenafil [7], bezafibrate [8], aspirin [9], and some traditional Chinese medicines [10, 11].

Naringin (4’, 5, 7-trihydroxy flavanone-7-rhamnoglucoside) is a flavanone glycoside of grapefruit and other citrus fruits that exhibits many pharmacological activities [12, 13]. Naringin inhibits both oxidative stress [14] and the inflammatory response [15] and reduces apoptosis [16]. Naringin is both neuroprotective [17] and hepatoprotective [18], and it promoted fracture healing in osteoporotic rats [19]. The etiopathogeneses of lung injuries and pulmonary fibrosis feature expression of TNF-α and transforming growth factor-β (TGF-β) [20]. Naringin may stimulate angiogenesis by activating VEGF expression [12, 19, 21]. However, to the best of our knowledge, no study has explored whether naringin improves the survival of random skin flaps. We explore this possibility in the present study.

## RESULTS

### General observations and survival of flaps

**Figure 1:**
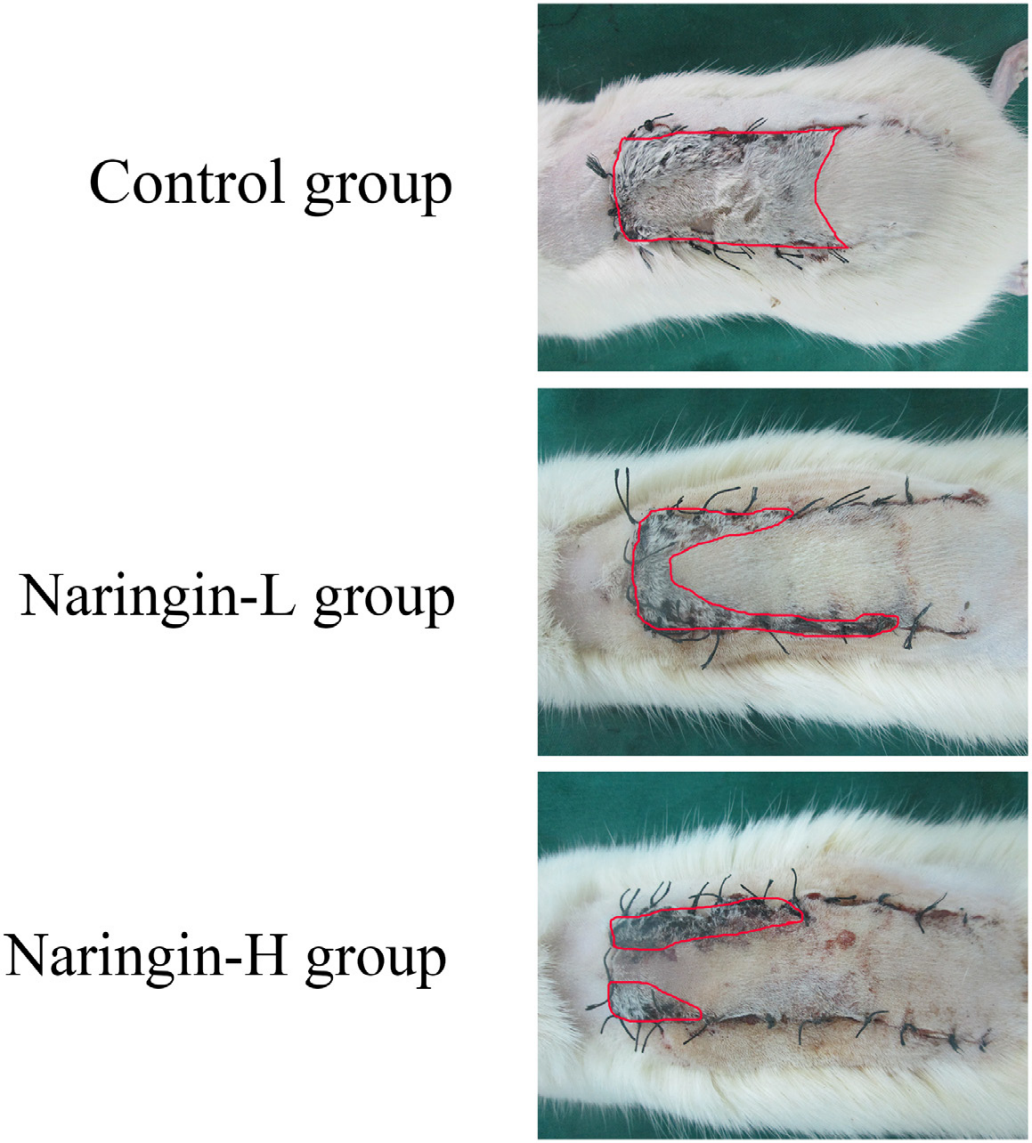
Digital photographs of flaps from the three groups and the necrosis parts were outlined by red line

Every flap was monitored by the same experimenter from day 1 to 7 postoperatively and the conditions (flap color, elasticity and necrosis) were recorded. On postoperative day 7, all groups showed well-demarcated borders between the surviving and necrotic parts; the necrotic areas presented dark, hard, and did not bleed when cut, while the surviving areas presented rosy, soft, and bled when cut (Figure 1).

**Figure 2:**
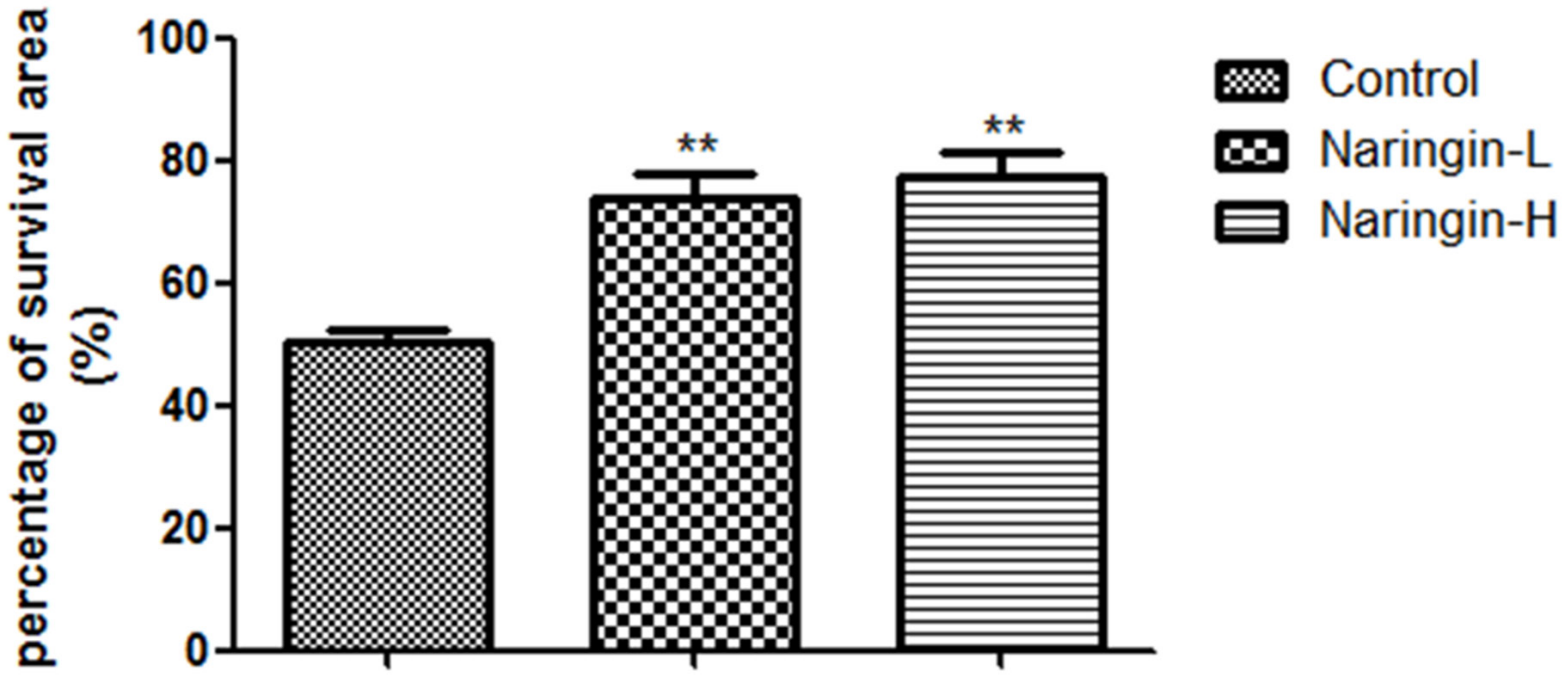
Percentage of survival area on day 7 in three groups (^**^p < 0.01 vs. the control group)

The flap survival portions were 73.89±4.01% in the Naringin-L group, 77.57±3.96% in the Naringin-H group and 50.2±2.23% in the controls (Figure 2). In the naringin group, flap survival area was significantly larger than the controls (^**^p < 0.01 vs. the control group).

### Histological changes

**Figure 3:**
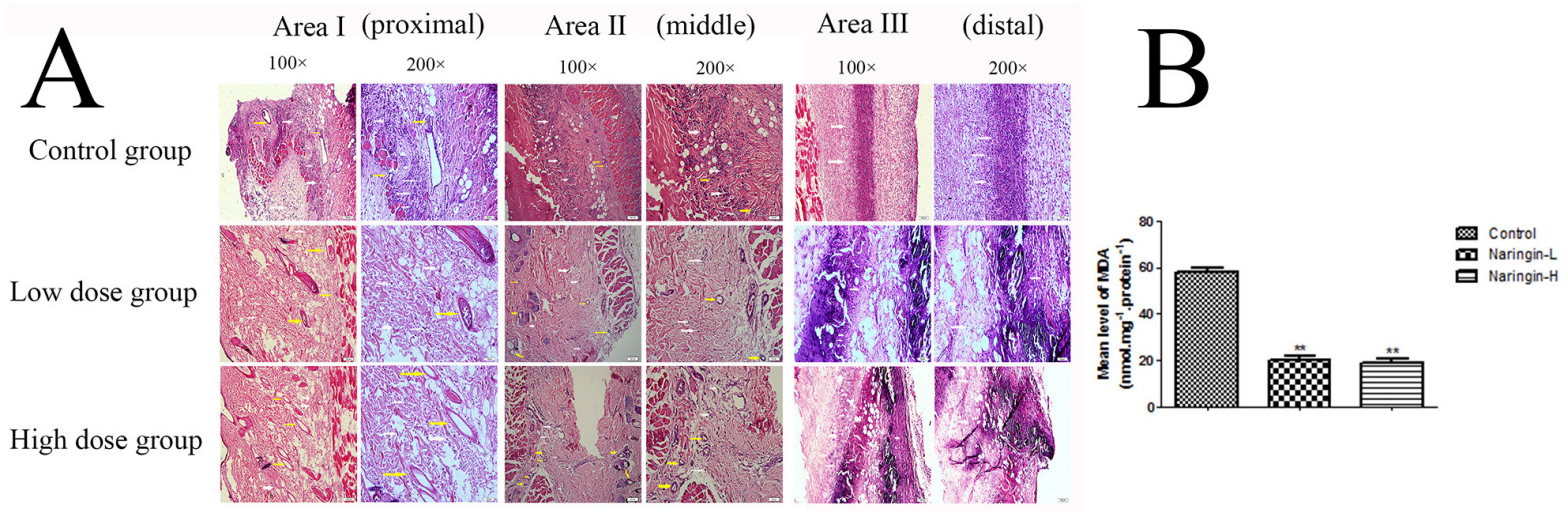
**(A)** Histological changes in the each area of the flaps in the three groups (100 and 200 magnification). **(B)** The MVDs of area II in the three groups (^**^p < 0.01 vs. the control group). Yellow arrow indicated the microvascular and white arrow indicated the neutrophil cells.

In order to evaluate the effects of naringin on the thickness of granulation tissue, tissue edema and infiltration of neutrophil cells, flap sections were prepared for HE staining. As demonstrated in Figure 3A, on day 7, the distal areas were morphologically similar in histological terms. All flaps exhibited similar changes in appearance; inflammatory cells infiltration was prominent, as were structural damage and edema. Ninety percent of tissue images revealed degeneration and necrosis of muscle fibers. Area II of the control group showed that many inflammatory cells were detected, while less in the treatment group, especially in the high-dose group, indicating that the inflammatory reaction was less severe in the treated flaps. Besides, the microvascular density (MVD) of area II was 33.38±2.90, 30.44±1.23, 14.04±2.66/mm2 in the Naringin-H groups, Naringin-L groups and control groups, respectively (Figure 3B; **p < 0.01 vs. the control group). In area I, the Naringin group exhibited edema, vascular dilation and inflammatory cells infiltration to a lesser extent than controls.

### Tissue edema

Percent tissue water content was significantly lower in the test group (low dose group: 52.66±3.48%; high dose group: 48.97±2.43%) compared with that in the control group (56.05± 2.13%) (p < 0.01).

### Flap angiography

**Figure 4:**
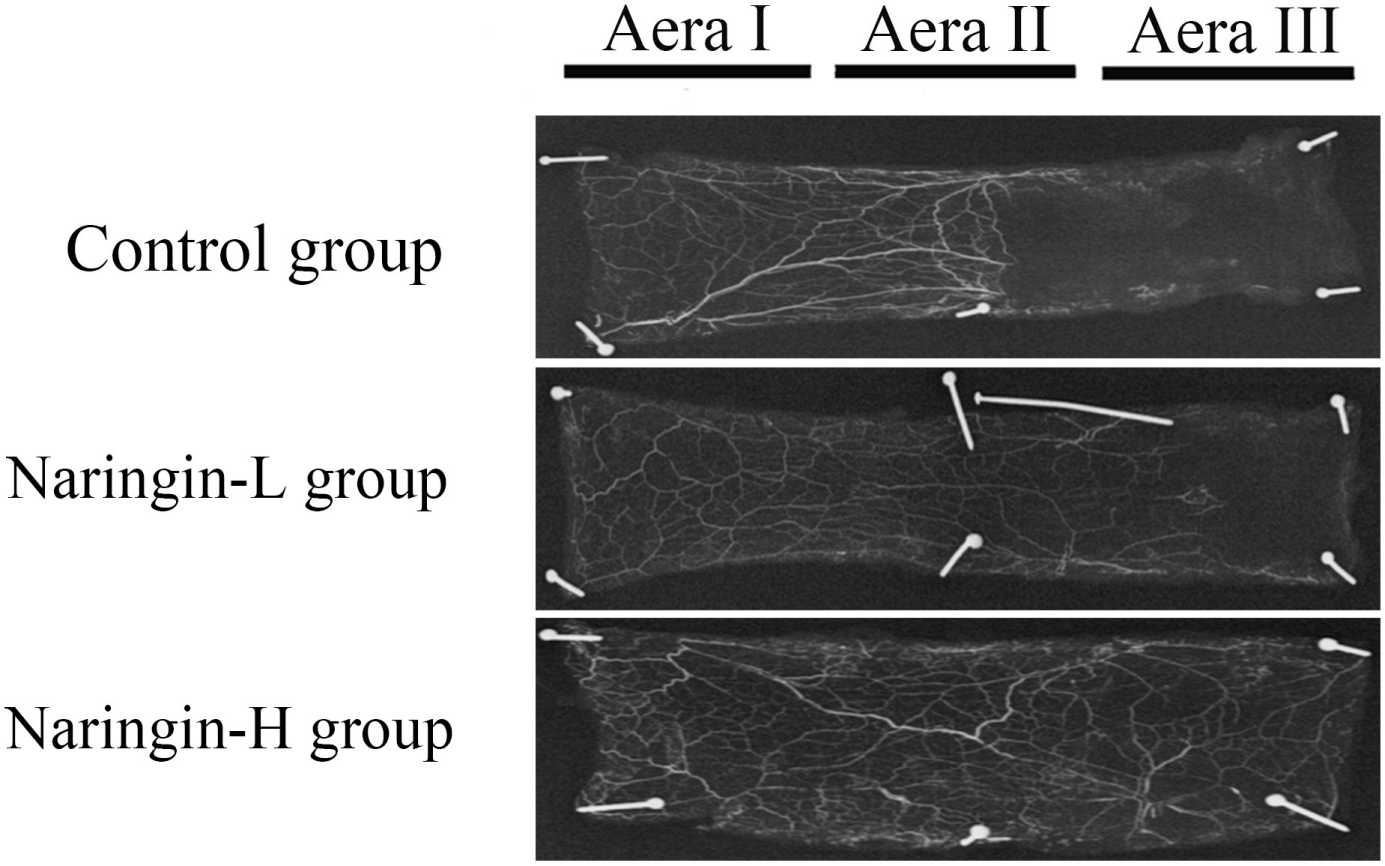
Flap angiography presenting flaps from the three groups

Through X-ray imaging obtained 7 days postoperatively, the microvascular of flaps were shown clearly. In the treatment group, particularly in the high-dose group the microvascular of flaps was greater than that in controls (Figure 4).

### VEGF expression

**Figure 5:**
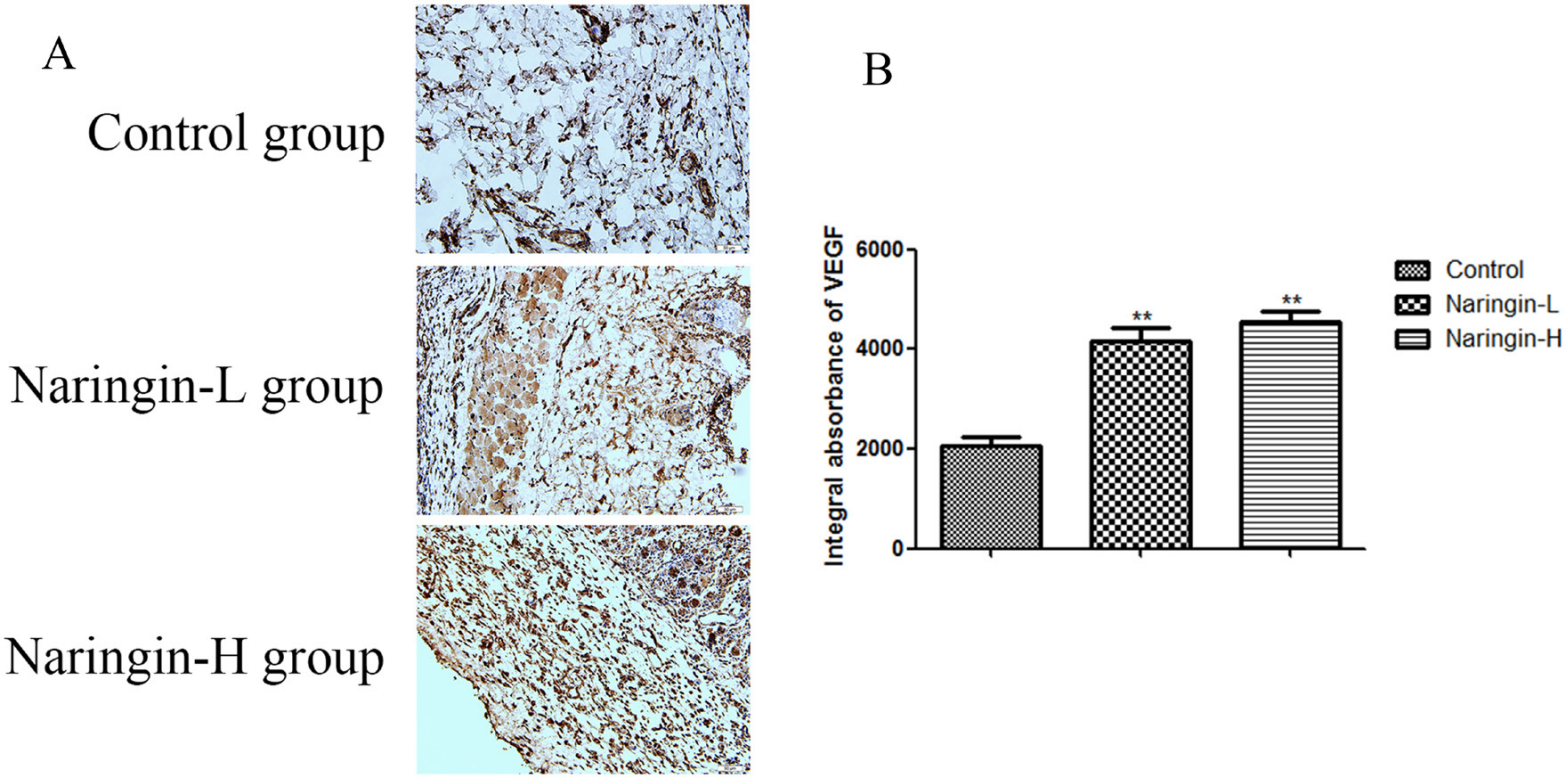
**(A)** Comparison of VEGF expression in the intermediate area II of the three groups. The immunohistochemistry test and observed under magnification 200. **(B)** IA value was detected to compare the level of VEGF (^**^p < 0.01 vs. the control group). IA, integral absorbance; VEGF, vascular endothelial growth factor.

The VEGF expression level in the naringin group was significantly higher than in the controls based on the IA value 4535±230 (Naringin-H), 4167±271 (Naringin-L), 2061±187 (Controls), respectively (Figure 5, **p < 0.01vs. the control group).

### Laser doppler imaging

**Figure 6:**
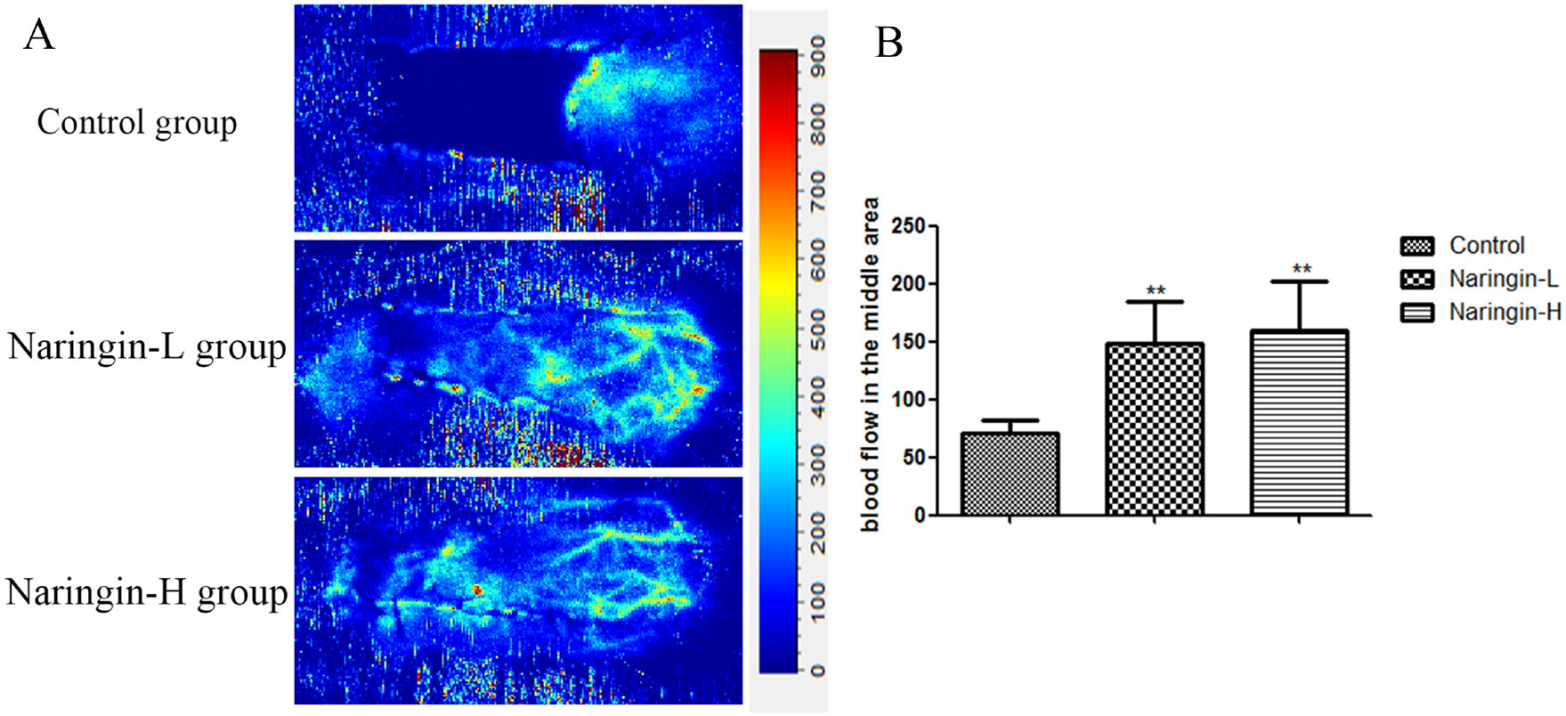
**(A)** The blood perfusion on day 7 in three groups. **(B)** Mean blood flow of the second choke zone in the flap in three groups measured by laser Doppler imaging at day 7 after surgery (^**^p < 0.01 vs. the control group).

On day 7, the Laser Doppler System was used to evaluate the blood flow of the flap. The blood flow of the middle area was 70.80±11.69 (Control group), 148.37±36.76 (Naringin-L), 159.98±42.67 (Naringin-H), respectively, and the blood flow in the treatment group was significantly larger than in the controls (Figure 6, **p < 0.01vs. the control group).

### Proinflammatory cytokines in serum

**Figure 7:**
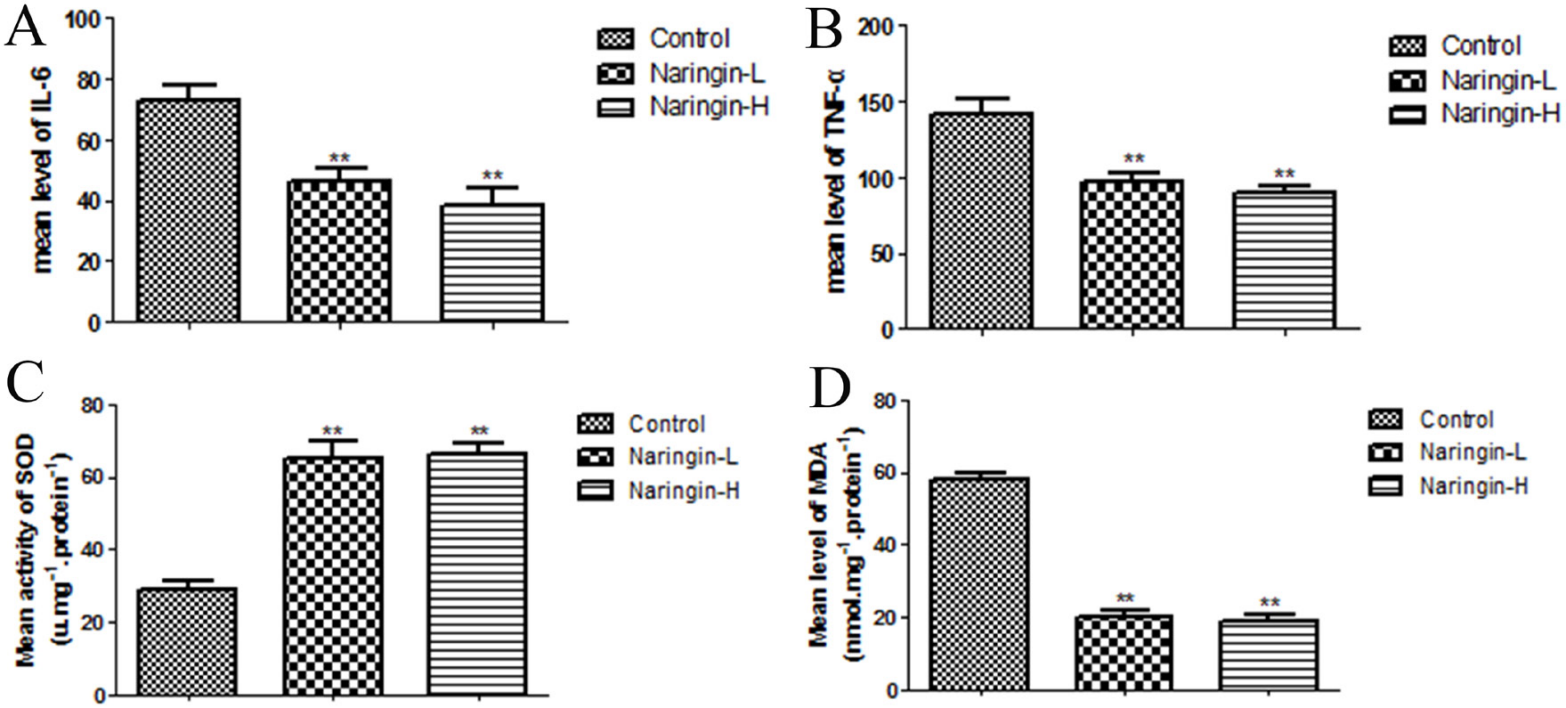
**(A, B)** The expression of the flap inflammation reaction factor (TNF-a, IL-6) (^**^p < 0.01 vs. the control group). **(C, D)** The expression of the flap oxidative stress factor (SOD, MDA) (^**^p < 0.01 vs. the control group).

The ELISA (enzyme linked immunosorbent assay) kits were used to explore the levels of proinflammatory cytokines such as IL-6 and TNF-α in serum, expressing the degree of inflammatory response. Naringin dramatically decreased the levels of IL-6 (46.16±4.39 pg/mL, low-dose group), (38.32±5.90 pg/mL, high-dose group) compared with the control group (72.67±5.04 pg/mL) (Figure 7A, **p < 0.01 vs. the control group), meanwhile the level of TNF-α after Naringin administration (96.85±6.39pg/mL, low-dose group), (89.44±4.83 pg/mL, high-dose group) was also reduced relative to the control group (142.16±8.75 pg/mL) (Figure 7B, **p < 0.01 vs. the control group). Summarily, these datas indicated Naringin had the ability of decreasing the release of cytokines in the flap.

### Superoxide dismutase activity and malondialdehyde content

The mean SOD activity level in the naringin group was 65.23±4.76 (Naingin-L), 66.32 ±2.87 (Naringin-H)U/mg protein, which was significantly higher than in the controls (28.80±2.33U/mg protein) (Figure 7C, **p < 0.01 vs. the control group). The mean MDA level in the naringin-L group (20.08±1.51 nmol/mg protein) and in the high group (18.71±1.71 nmol/mg protein) was significantly lower than that in the controls (58.24±1.64 nmol/mg protein) (Figure 7D, **p < 0.01 vs. the control group).

## DISCUSSION

Acceptable functional restoration and good cosmetic results are among the desired endpoints of tumor resection. Flap placement may afford satisfactory esthetic and functional outcomes and is increasing in popularity. However, distal flap necrosis attributable to ischemia is a major problem, increasing treatment costs and in-hospital time. Ischemia, ischemia–reperfusion injury, and inflammation may all be in play [22–25]. We found that naringin protected against flap necrosis by inhibiting apoptosis, attenuating the inflammatory response and oxidative stress, and stimulating blood vessel growth.

VEGF, a multifunctional growth factor, plays a central role in angiogenesis, promoting neovascularization and endothelial cell proliferation, which in turn improve flap survival [26, 27]. Angiogenesis greatly enhances vasculogenesis by increasing the delivery levels of oxygen, nutrients, and collagen building blocks [28]. Earlier, naringin was found to trigger VEGF synthesis, to enhance angiogenesis, and to improve capillary density in rats with diabetic foot ulcers. The angiogenetic effect was especially marked [12, 29]. We found that, compared with control rats, the IA (integral absorbance) value was markedly increased in the naringin groups, as were the mean vessel density in area II and the vessel numbers revealed by flap angiography and laser Doppler imaging. However, although VEGF expression was greater in the high-dose than in the low-dose naringin group, the improvements in flap survival were similar between the two groups. Naringin indeed promoted angiogenesis, increasing vessel numbers in damaged tissue by enhancing blood supply and VEGF expression. However, angiogenesis mediated by VEGF is a double-edged sword, promoting flap survival on the one hand and facilitating tumor metastasis on the other. Therefore, the use of VEGF-enhancing drugs requires further investigation.

The pro-inflammatory growth factor TNF-α facilitates release of the inflammatory mediators interleukin (IL)-1, IL-6, and IL-8, aggravating tissue damage and triggering apoptosis. Recently, VEGF was shown to significantly reduce TNF-α expression and inflammation, both in flaps and other ischemic tissues [26, 30]. Inflammation greatly compromises flap survival [31]. The level of MDA (a major marker of lipid peroxidation) directly reflects the extent of tissue injury [32]. SOD, a crucial endogenous anti-oxidase, protects cells from injury caused by toxic oxygen-derived free radicals, and the SOD level is a sensitive indicator of antioxidant status [33]. We found that the oxidative stress level and serum levels of the pro-inflammatory cytokines TNF-α and IL-6 were significantly reduced in the naringin groups compared with the control, suggesting that naringin alleviated inflammation and oxidative stress, thus effectively preventing ischemia–reperfusion injury. The detailed mechanism involved requires further study.

Thus, naringin improved flap survival by inhibiting inflammation and oxidative stress and promoting angiogenesis. In clinical practice, naringin may enhance flap survival. Additional clinical and experimental studies are required.

## MATERIALS AND METHODS

### Animal

We obtained 90 male SD rats (weight, 250–300g) from Wenzhou Medical University (SYXK [Zhe] 2015–0009). All experimental procedures were approved by the Research Ethics Committee of Wenzhou Medical University.

### Reagents

Naringin was purchased from Sigma Chemical Co. (St Louis, MO, USA). Anti-VEGF (anti–vascular endothelial growth factor) polyclonal antibody (pAb) was obtained from Bioworld (Nanjing, China). SOD and MDA test kits were purchased from Nanjing Jiancheng Biology Institution (Nanjing, China). Rat TNF- α and IL-6 ELISA kits were purchased from Nanjing KeyGEN Biotech (Nanjing, China).

### Flap animal model and drug administration

**Figure 8:**
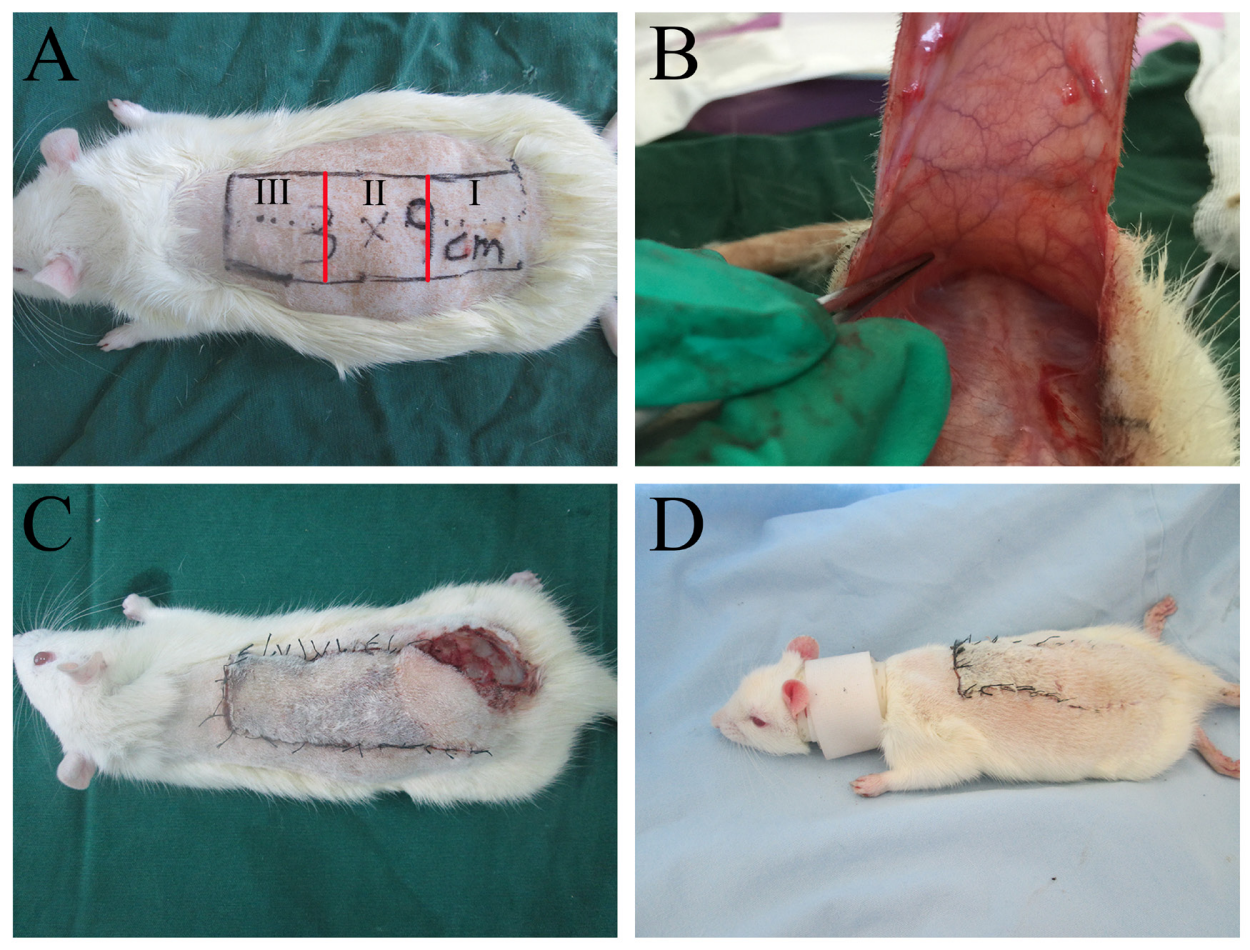
**(A)** A McFarlane flap model was designed (3×9 cm) and three parts were divided (I, II, and III); **(B)** no axial vessels were incorporated into the flap; **(C)** the rats had a self-mutilation tendency and they would bite the flaps on their back and cause flap injuries; **(D)** the rats were fitted with a neck collar to prevent self-mutilation.

The modified McFarlane flap model (3 × 9cm) was applied to every rat [34]. The area of the dorsum was divided into three equal segments: I, II and III (proximal, intermediate and distal) (Figure 8). Following the surgical procedure, all 90 rats were randomly divided into three groups: a 40 mg/kg naringin-treated group (n = 30, low-dose group); an 80 mg/kg naringin-treated group (n = 30, high-dose group) and a 40 mg/kg control group (0.5 % sodium carboxymethylcellulose) (n = 30). For oral administration, naringin was suspended in 0.5 % sodium carboxymethylcellulose. All rats were treated for 7 days. Postoperatively, all rats were housed in an environmentally controlled room, in standard experimental cages individually and fed standard rat chow and water ad libitum. Because of the self-mutilation, all rats were tied with a collar [35].

### Flap survival measurement

The surviving area of each flap was observed macroscopically during the injection and the appearance, tissue elasticity and hair condition were assessed. After 7 days, we measured the surviving area and total area of each flap using transparent cellophane and the weights of the flap using an electronic scale; the surviving area was calculated as a percentage [8]. The following formula was used to quantify the results: extent of the viable area × 100/the total area (viable and ischemic) [24].

### Histological staining

After all rats were sacrificed, three samples (size in 1×1cm) of the centre flap tissue were harvested from each area, fixed in 4% paraformaldehyde, and routine procedures were performed. Paraffin blocks were cut into 4 μm slices, and stained with hematoxylin and eosin. The Olympus BH51 light microscope (Olympus Corporation, Tokyo, Japan; ×100 magnification) was used to evaluate the condition of tissue edema, the thickness of granulation tissue and count the number of neutrophil cells. Furthermore, in order to comprehend the condition of microvascular density, we also calculated the microvessel number in each unit area(/mm^2^).

### Tissue edema measurements

After executing the rats, we weighed flap tissue using an electronic scale, and dehydrated using an autoclave with the temperature of 50°C. In order to obtain a constant weight, all samples were weighed daily and until the weight did not change for 2 days. Water content reflected the degree of edema. The percent water content was determined by the following equation: Tissue% water content = ([wet weight-dry weight]/ wet weight)×100%.

### Flap angiography

Using a 24-gauge intravenous catheter based on a modified lead oxide-gelatin inoculability tip, ten rats choosing from every group sustained whole-body angiography at 7 days after the operation [36]. Heparin saline (1%, 1.5 mL) was injected to the right carotid artery and then the contrast medium (150 mL/kg) consisted of water, lead oxide and gelatin, was followed. After fixation overnight, we radiographed the flap using an X-ray machine (54 kVp, 40 mA, 100-second exposure).

### Laser doppler imaging

All animals were performed using a laser Doppler imaging system (Lisca PIMII, Stockholm, Sweden) on the day of sacrifice. The blood perfusion monitor (PeriFlux System 5000; PERIMED, Järfälla, Sweden) began one hour after stabilization. The second choke zone was inspected for 1 minute. The mean blood flow was expressed as perfusion units [37].

### Immunohistochemical staining

Immunohistochemical staining was conducted for VEGF using the streptavidin-peroxidase method [38]. Flap tissues were imaged at ×400 magnification using a DP2-TWAN image-acquisition system (Olympus Corp., Tokyo, Japan). Images were saved using Image-Pro Plus software, version 6.0 (Media Cybernetics, Rockville, MD) and the VEGF expression levels was detected by the IA values.

### SOD activity and mda content

Ten tissue samples (5 × 5 mm) were gained from Areas II at 7 days after the operation, and then a series of procedures were carried out including weighing, homogenizing and diluting. SOD activity was measured by the oxidase enzymatic method, and MDA level was gauged through an approach triggered by a reaction with thiobarbituric acid at 90 to 100°C [39].

### Determination of cytokines in serum by ELISA

According to the manufacturer’s instructions, the ELISA kits were applied to measure the levels of IL-6 and TNF-α.

### Statistical analysis

The SPSS software (ver.20.0; SPSS Inc., Chicago, IL) was used to statistical analyses and all values were marked as mean ± SD. Multiple comparisons between groups were using one-way ANOVA and Tukey’s post hoc test. P values < 0.05 were considered to indicate statistical significance.

## Abbreviations

VEGF: vascular endothelial growth factor
SOD: measuring superoxide dismutase
MDA: malondialdehyde
TNF-α: tumor necrosis factor-α
TGF-β: transforming growth factor-β
MVD: microvascular density
HE: hematoxylin and eosin
IA: integral absorbance
IL-1: interleukin-1
IL-6: interleukin-6
IL-8: interleukin -8
ELISA: enzyme linked immunosorbent assay
ANOVA: analysis of variance
